# Early assessment of shear wave elastography parameters foresees the response to neoadjuvant chemotherapy in patients with invasive breast cancer

**DOI:** 10.1186/s13058-021-01429-4

**Published:** 2021-04-29

**Authors:** Juanjuan Gu, Eric C. Polley, Max Denis, Jodi M. Carter, Sandhya Pruthi, Adriana V. Gregory, Judy C. Boughey, Robert T. Fazzio, Mostafa Fatemi, Azra Alizad

**Affiliations:** 1 Department of Physiology and Biomedical Engineering, Mayo Clinic College of Medicine and Science, 200 First Street SW, Rochester, MN 55905 USA; 2 Department of Health Science, Mayo Clinic College of Medicine and Science, Rochester, MN 55905 USA; 3 Department of Radiology, Mayo Clinic College of Medicine and Science, Rochester, MN 55905 USA; 4 Department of Laboratory Medicine & Pathology, Mayo Clinic College of Medicine and Science, Rochester, MN 55905 USA; 5 Department of Medicine, Mayo Clinic College of Medicine and Science, Rochester, MN 55905 USA; 6 Department of Surgery, Mayo Clinic College of Medicine and Science, Rochester, MN 55905 USA

**Keywords:** Immunohistochemical biomarkers, Ki-67, Shear wave elastography, Neoadjuvant chemotherapy, Breast cancer, Mass characteristic frequency

## Abstract

**Background:**

Early prediction of tumor response to neoadjuvant chemotherapy (NACT) is crucial for optimal treatment and improved outcome in breast cancer patients. The purpose of this study is to investigate the role of shear wave elastography (SWE) for early assessment of response to NACT in patients with invasive breast cancer.

**Methods:**

In a prospective study, 62 patients with biopsy-proven invasive breast cancer were enrolled. Three SWE studies were conducted on each patient: before, at mid-course, and after NACT but before surgery. A new parameter, mass characteristic frequency (*f*_mass_), along with SWE measurements and mass size was obtained from each SWE study visit. The clinical biomarkers were acquired from the pre-NACT core-needle biopsy. The efficacy of different models, generated with the leave-one-out cross-validation, in predicting response to NACT was shown by the area under the receiver operating characteristic curve and the corresponding sensitivity and specificity.

**Results:**

A significant difference was found for SWE parameters measured before, at mid-course, and after NACT between the responders and non-responders. The combination of *E*_mean2_ and mass size (*s*_2_) gave an AUC of 0.75 (0.95 CI 0.62–0.88). For the ER+ tumors, the combination of *E*_mean_ratio1_, *s*_1_, and Ki-67 index gave an improved AUC of 0.84 (0.95 CI 0.65–0.96). For responders, *f*_mass_ was significantly higher during the third visit.

**Conclusions:**

Our study findings highlight the value of SWE estimation in the mid-course of NACT for the early prediction of treatment response. For ER+ tumors, the addition of Ki-67improves the predictive power of SWE. Moreover, *f*_mass_ is presented as a new marker in predicting the endpoint of NACT in responders.

## Introduction

Neoadjuvant chemotherapy (NACT) is an established therapeutic strategy for operable breast cancers and locally advanced breast cancers and allows more patients to undergo breast-preserving surgery [1, 2]. A pathological complete response (pCR) to NACT is associated with increased disease-free interval. However, responses to NACT are quite variable. With the increased use of NACT, it is crucial to have an accurate prediction of tumor response to NACT.

Current techniques available for monitoring response to NACT are positron emission tomography (PET) [3], sonography, mammography, magnetic resonance imaging (MRI) [4–7], and shear wave elastography (SWE) [8, 9]. Conventional sonography and mammography have poor reliability in evaluating the size of residual tumor after chemotherapy [10]. SWE is a recently developed low-cost imaging technique for measuring tissue stiffness in a noninvasive and quantitative manner with high reproducibility [11–15].

Tissue stiffness has been demonstrated to be significantly correlated with tumor growth as cancer development and progression require extensive reorganization of the extracellular matrix (ECM) [16]. Increased deposition of collagen and other ECM molecules enhances the stiffness of tumoral stroma [17–19]. Changes in tumor stiffness were significantly greater in patients who had a good response to NACT compared to those resistant to NACT [20]. Breast cancer pre- and post-treatment stiffness obtained from SWE was significantly correlated with the presence of residual cancer [8, 9]. A study in [21] showed that the SWE stiffness measured after 3 cycles of NACT and changes in stiffness from baseline were strongly associated with pCR after 6 cycles. The combination of the post-treatment SWE and greyscale ultrasound has also been shown to be promising for end-of-treatment identification of residual disease and thus response to NACT, with similar accuracies found in assessment by MRI [22].

In the current study, a new SWE parameter mass characteristic frequency (*f*_mass_) was used. *f*_mass_ is defined as the ratio of the averaged minimum shear wave speed (SWS) within the regions of interest (ROIs) to the largest mass dimension. The physical meaning of the new parameter can be explained as the inverse of the maximum shear wave propagation time in a breast mass. The motivation for using *f*_mass_ is that in SWE, the SWS of small masses is often underestimated due to their small size compared to the wavelength. This error may lead to the false-negative diagnosis of such masses. *f*_mass_ represents the SWS weighted by the inverse of mass diameter; therefore, *f*_mass_ assumes a larger value for masses that are too small. Thus, one may expect *f*_mass_ to be a more robust parameter in SWE than SWS itself. However, we want to emphasize that *f*_mass_ is not meant to compensate for the underestimation error of SWS in a mathematical sense. Our intention is to introduce *f*_mass_ as a new metric that improves the characterization of breast masses in a statistical sense. The purpose of this study was to investigate the role of SWE parameters, including mass characteristic frequency, in evaluating the breast tumor response to the NACT treatment. For ER-positive tumors, the combination of the SWE parameters with Ki-67 was further studied to improve the sensitivity and specificity of the response prediction.

## Methods

### Study population

This prospective study was Health Insurance Portability and Accountability Act (HIPAA) compliant and was approved by the Institutional Review Board (IRB) (IRB application #12-003329). From January 2014 to September 2020, 62 female patients (age range 27–78 years) with 62 biopsy-proven invasive breast cancers were recruited in this study. During the recruitment, patients with prior mastectomy or breast implant were excluded. One patient with a previous lumpectomy in the contralateral breast was included in this study. A signed written informed consent with permission for publication was obtained from each enrolled patient prior to the study.

### Imaging

**Fig. 1:**
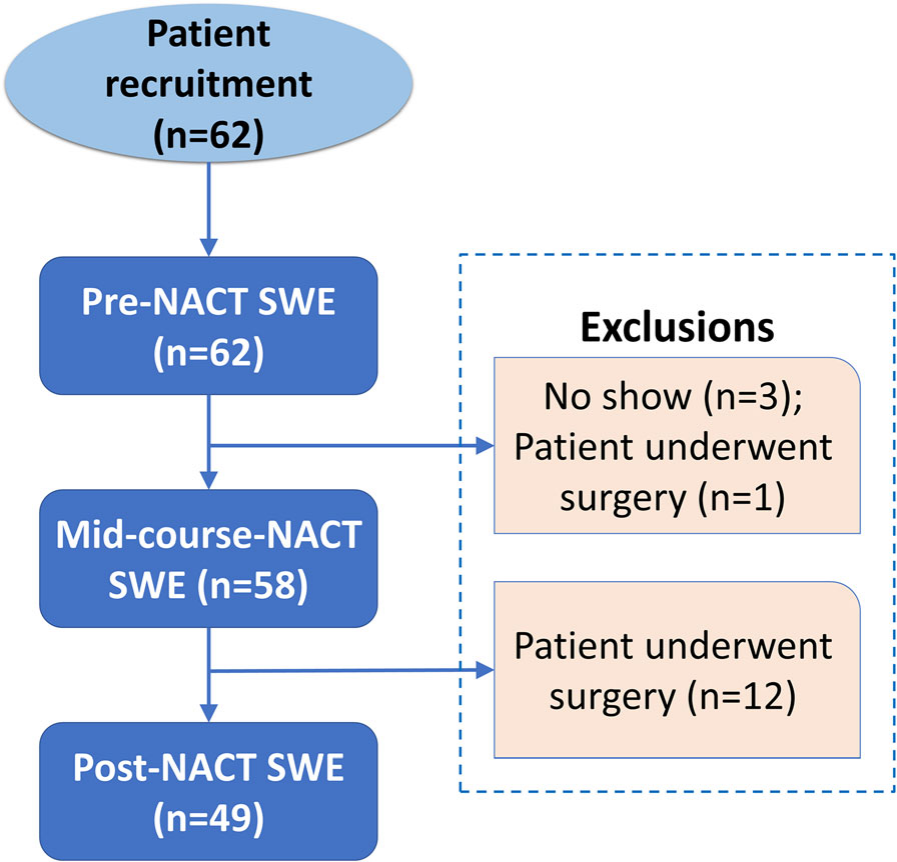
Flow diagram of the study population. For the pre-NACT SWE study, only 56 patient results were analyzed because the first 6 patients were scanned with a different ultrasound machine. NACT, neoadjuvant chemotherapy; SWE, shear wave elastography

SWE studies were conducted for each patient at three time points: before initiation of NACT, at the mid-course of NACT, and after completion of NACT but prior to surgery. A flow diagram of the study population is summarized in Fig. 1.

**Fig. 2:**
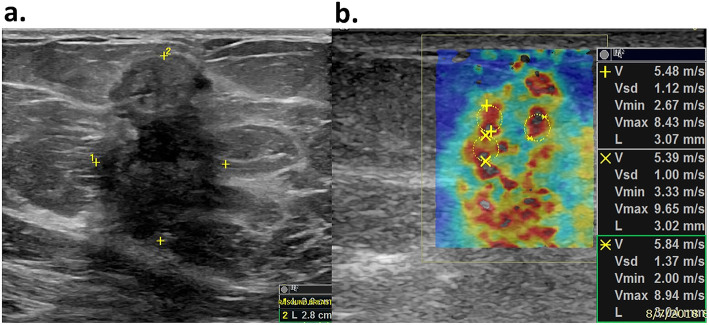
An example for calculating the *f*_mass_ for a 59-year-old female patient with grade II invasive ductal carcinoma. **a** The mass size *d* was read as the greatest dimension shown in the clinical B-mode image, and it is 28 mm in this example. **b** The minimum shear wave speed was calculated as the average value of the minimum shear wave speed from the three ROIs shown in the SWE image and is 2.7 m/s. Therefore, the *f*_mass_ for this measurement is 96 Hz. ROI, region of interest; SWE, shear wave elastography

The 2D SWE scanning was performed by one of our two experienced sonographers, using the GE LOGIQ-E9 ultrasound clinical scanner equipped with a 2–8 MHz linear array probe (9L-D, GE Healthcare, Wauwatosa, WI) for both the conventional B-mode and SWE data acquisition. To reduce motion artifacts, patients were instructed to suspend respiration for approximately 3 s during the data acquisition. The SWE measurement was acquired within a rectangle-shaped field of view, which covered the whole lesion and the adjacent normal tissue. For each lesion, along the same orientation, at least four SWE images were obtained. One of the consistent stiffness maps was chosen to draw ROIs. Three non-overlapping ROIs, 3 mm in diameter, were placed at the stiffest position of the lesion, with peritumoral stroma included. One ROI was placed at the surrounding normal tissue. The mean SWS, maximum SWS, minimum SWS, and standard deviation of the SWS inside each ROI were calculated by the ultrasound machine. The SWS for the tumor was represented by the average values of the three ROIs placed at the stiffest position. A new shear wave parameter, mass characteristic frequency, represented by *f*_mass_, is used in this paper: *f*_mass_ = 1000*V*_min_*/d*, where *f*_mass_ is with unit Hz, *V*_min_ is the minimum SWS with unit m/s, and *d* is the mass size in mm and recorded as the maximum dimension of the tumor shown on the B-mode image. Figure 2 illustrates the measurements for calculating *f*_mass_.

### Clinical pathologic data

The parameters for residual cancer burden (RCB) measurement for each patient were obtained from the surgical pathology report, and the RCB score was calculated with an empirical equation: RCB = 1.4(*f*_inv_*d*_prim_)^0.17^ + [4(1–0.75^LN^)*d*_met_]^0.17^ [23]. RCB was on a continuous scale and was further categorized as 0 (RCB = 0), I (0 < RCB < =1.36), II (1.36 < RCB < =3.28), or III (RCB > 3.28). Categories 0 and I were regarded as responders while categories II and III were regarded as non-responders [23, 24]. Of the 62 patients included in this study, 60 underwent lumpectomy or mastectomy, and the corresponding RCB scores were calculated from the surgical reports. Two patients did not undergo surgery, one progressed on neoadjuvant chemotherapy and died before undergoing surgery, and one developed metastatic disease, and therefore, surgery was not indicated and ultimately died 3.5 years after diagnosis. The two deceased patients were categorized as non-responders. The MRI dimensions (AP, trans, and SI) were read from the clinical MRI image that was acquired close to the date of the SWE scanning. The MRI volume was calculated as the product of the three dimensions (volume = AP × trans × SI). The clinical MRI was based on the dynamic enhanced protocol using intravenous (IV) contrast administration. Obtained T1- and T2-weighted images were analyzed with computer-aided detection (CAD) image analysis. With the help from a radiologist, the maximum lesion size was read from the B-mode imaging.

Estrogen receptor (ER), progesterone receptor (PR), human epidermal growth factor receptor 2 (HER2), and Ki-67 proliferative index status of the pre-NACT tumor needle core biopsies were obtained from the clinical record. ER and PR were considered negative if less than 1% of invasive tumor cells were immunoreactive, and were considered positive if greater than or equal to 1% of invasive tumor cells were immunoreactive. As per the American Society of Clinical Oncology (ASCO)/College of American Pathologists (CAP) guidelines [25], immunohistochemical HER2 scores or of 0 and 1+ were considered negative and a score of 3+ was scored as positive. Equivocal HER2 immunostains (HER2 scores of 2+) underwent fluorescence in situ hybridization testing for HER2 amplification and were classified as per the ASCO/CAP guidelines. Ki-67 immunostain was reported as a percentage of positively staining nuclei.

Based on the clinical biomarker data, the tumors were divided into five molecular subtypes according to the St. Gallen criteria [26]: Luminal A—ER positive, PR positive/negative, HER2 negative, and Ki-67 < 14%; Luminal B (HER2−)—ER positive, PR positive/negative, HER2 negative, and Ki-67 ≥ 14%; Luminal B (HER2+)—ER positive, PR positive/negative, HER2 positive, and any Ki-67; HER2 positive—ER negative, PR negative, and HER2 positive; and triple-negative (TN)—ER negative, PR negative, and HER2 negative. Among them, Luminal A, Luminal B (HER2−), and Luminal B (HE2+) types were ER-positive tumors.

### Statistical analysis

The measured SWS was converted to elasticity expressed in kilopascals [27]. Changes of SWE parameters were also calculated:

$$ {E}_{\mathrm{mean}1-2}={E}_{\mathrm{mean}1}-{E}_{\mathrm{mean}2}, $$

$$ {E}_{\mathrm{mean}1-3}={\mathrm{E}}_{\mathrm{mean}1}-{E}_{\mathrm{mean}3}, $$

$$ {E}_{\max 1-2}={E}_{\mathrm{max}1}-{\mathrm{E}}_{\mathrm{max}2}, $$

$$ {E}_{\max 1-3}={E}_{\mathrm{max}1}-{E}_{\mathrm{max}3}, $$

$$ {f}_{\mathrm{mass}1-2}={f}_{\mathrm{mass}1}-{f}_{\mathrm{mass}2}, $$

$$ {f}_{\mathrm{mass}1-3}={f}_{\mathrm{mass}1}-{f}_{\mathrm{mass}3}. $$

The subscripts 1, 2, and 3 indicate the corresponding parameters measured at the first, the second, and the third visits, respectively.

Statistical analysis was conducted with RStudio (RStudio, PBC, Boston, MA). The Kruskal-Wallis test and Pearson’s chi-squared test were used in the statistical analysis to calculate the *p* value for continuous data and count data, respectively. *p* < 0.05 was considered indicative of a statistically significant difference. Leave-one-out cross-validation (LOOCV) [28] was used to assess the effect of multiple factors on the prediction of the response to NACT. Receiver operating characteristic (ROC) curve analysis was used to calculate the area under the curve (AUC) and determine the cutoff values, as well as the corresponding sensitivity and specificity. The optimal cutoff was defined as the point closest to the point (0, 1) on the ROC curve.

## Results

### Clinical parameters during the NACT

Patient demographic and tumor characteristics of the 62 patients enrolled in this study are presented in Table 1. Overall, as expected, compared to other histologic subtypes, patients with invasive ductal carcinoma had higher rates of response to NACT (*p* = 0.03), and higher tumor grade (grade III) had a higher response rate of 67.7% compared to 32.3% to lower grade tumors (grade I/II). Among different ER-positive molecular subtypes, a significant difference was found for the response rate (*p* = 0.03) with the highest response rate seen in Luminal B (HER2+) type cancers. Table 2 summarizes the MRI volume (*V*_MRI_), mass size (*s*), and the SWE parameters, including the new parameter *f*_mass_. The averaged MRI volume, shear wave elasticity, and mass size decreased during the NACT treatment for both the responder group and the non-responder group. Tumor response was significantly correlated with the values of *E*_mean-ratio1_ measured during the first SWE visit; *s*_2_, *E*_mean2_, and *E*_max2_ measured during the second SWE visit; and *V*_MRI3_, *s*_3_, *E*_mean3_, *E*_max3_, *E*_mean-ratio3_, *E*_max-ratio3_, and *f*_mass3_ measured during the third SWE visit.

**Table 1:** Participant demographics and clinical pathological results

Parameters	Responder (*n* = 34)	Non-responder (*n* = 28)	*p* value
Age at enrollment (years)^a^	52.8 ± 11.3	53.1 ± 16.3	0.72
Pathologic type			0.03*
Invasive ductal carcinoma	29 (63.0)	17 (37.0)	
Invasive lobular carcinoma	4 (50.0)	4 (50.0)	
Invasive mammary carcinoma with mixed ductal and lobular features	1 (12.5)	7 (87.5)	
Pathologic grade			0.05
I/II	11 (39.3)	17 (60.7)	
III	23 (67.7)	11 (32.3)	
Estrogen receptor status			0.17
Positive	20 (47.6)	22 (52.4)	
Negative	14 (70.0)	6 (30.0)	
Progesterone receptor status			0.44
Positive	20 (50.0)	20 (50.0)	
Negative	14 (63.6)	8 (36.4)	
HER2 status			0.19
Positive	15 (68.2)	7 (31.8)	
Negative	19 (47.5)	21 (52.5)	
Ki-67 index (%)			
Measured^a^	40.1 ± 25.1 (46.7)	27.0 ± 20.1 (53.3)	0.05
Data missing	(76.5)	(23.5)	
Subtype			
ER+ tumor types			0.03*
Luminal A	1 (12.5)	7 (87.5)	
Luminal B (HER2-)	8 (47.1)	9 (52.9)	
Luminal B (HER2+)	11 (68.8)	5 (31.2)	
Other tumor types			0.92
HER2+	4 (66.7)	2 (33.3)	
TN	9 (81.8)	2 (18.2)	
Data missing	1 (25.0)	3 (75.0)	

Data in parentheses are percentages

*ER* estrogen receptor, *HER2* human epidermal growth factor receptor 2, *TN* triple-negative

^a^Data are mean ± standard deviation

*p < 0.05; the difference is statistically significant

**Table 2:** Summary of the clinical MRI and SWE parameters for the 62 patients

Parameters	Responder (*n* = 34)	Non-responder (*n* = 28)	*p* value
MRI volume (mm^3^)			
*V*_MRI1_	104,589.2 ± 254,548.7 (24)	117,407.7 ± 164,022.0 (25)	0.17
*V*_MRI2_	24,458.6 ± 65,700.82 (16)	115,921.4 ± 131,323.6 (7)	0.09
*V*_MRI3_	16,939 ± 52,964.8 (14)	72,912.7 ± 123,127.2 (14)	0.04*
Mass size (mm)			
*s*_1_	27.1 ± 13.7 (28)	34 ± 20.5 (28)	0.27
*s*_2_	16.5 ± 8.5 (32)	29.0 ± 16.5 (26)	< 0.001*
*s*_3_	10.6 ± 6.6 (25)	24.3 ± 15.4 (24)	< 0.001*
Mean elasticity (kPa)			
*E*_mean1_	82.2 ± 38.0 (28)	92.1 ± 32.9 (28)	0.76
*E*_mean2_	38.5 ± 22.7 (32)	60.0 ± 30.3 (26)	0.01*
*E*_mean3_	29.5 ± 25.4 (25)	47.6 ± 33.3 (24)	0.03*
Maximum elasticity (kPa)			
*E*_max1_	163.1 ± 55.7 (28)	179.6 ± 38.6 (28)	0.50
*E*_max2_	95.7 ± 51.1 (32)	134.9 ± 59.0 (26)	0.01*
*E*_max3_	63.4 ± 49.8 (25)	114.3 ± 72.9 (24)	0.02*
Ratio of mean elasticity			
*E*_mean-ratio1_	16.2 ± 14.8 (28)	26.0 ± 21.2 (28)	0.02*
*E*_mean-ratio2_	10.9 ± 10.2 (32)	16.8 ± 14.9 (26)	0.07
*E*_mean-ratio3_	5.3 ± 6.1 (25)	10.8 ± 9.8 (24)	0.02*
Ratio of maximum elasticity			
*E*_max-ratio1_	16.6 ± 14.0 (28)	22.2 ± 14.0 (28)	0.08
*E*_max-ratio2_	14.3 ± 15.1 (32)	23.0 ± 31.8 (26)	0.22
*E*_max-ratio3_	6.1 ± 5.5 (25)	15.6 ± 16.2 (24)	0.02*
Mass characteristic frequency (Hz)			
*f*_mass1_	125.7 ± 71.2 (28)	119.1 ± 74.8 (28)	0.82
*f*_mass2_	135.3 ± 72.2 (32)	105.0 ± 79.2 (26)	0.05
*f*_mass3_	243.9 ± 156.3 (25)	102.2 ± 61.2 (24)	< 0.001*
Change of elasticity (kPa)			
*E*_mean1–2_	39.8 ± 35.9 (26)	31.3 ± 42.4 (26)	0.42
*E*_mean1–3_	50.9 ± 41.4 (19)	45.6 ± 43.1 (24)	0.46
*E*_max1–2_	57.2 ± 60.8 (26)	48.5 ± 69.7 (26)	0.50
*E*_max1–3_	102.7 ± 56.0 (19)	65.8 ± 77.3 (24)	0.09
Change of mass characteristic frequency (Hz)			
*f*_mass1–2_	−8.9 ± 97.6 (26)	16.1 ± 86.3 (26)	0.90
*f*_mass1–3_	−88.4 ± 98.4 (19)	16.1 ± 55.4 (24)	< 0.001*

Data are mean ± standard deviation; data in parentheses are mass numbers

*E*_*mean*_ mean elasticity, *E*_*max*_ maximum elasticity, *E*_*mean_ratio*_ ratio of the mean elasticity between the mass and the surrounding normal tissue, *E*_*max_ratio*_ ratio of the maximum elasticity between the mass and the surrounding normal tissue, *f*_*mass*_ mass characteristic frequency

The subscripts 1, 2, and 3 indicate the corresponding parameters measured at the first, the second, and the third visit, respectively

*p < 0.05; the difference is statistically significant

**Fig. 3:**
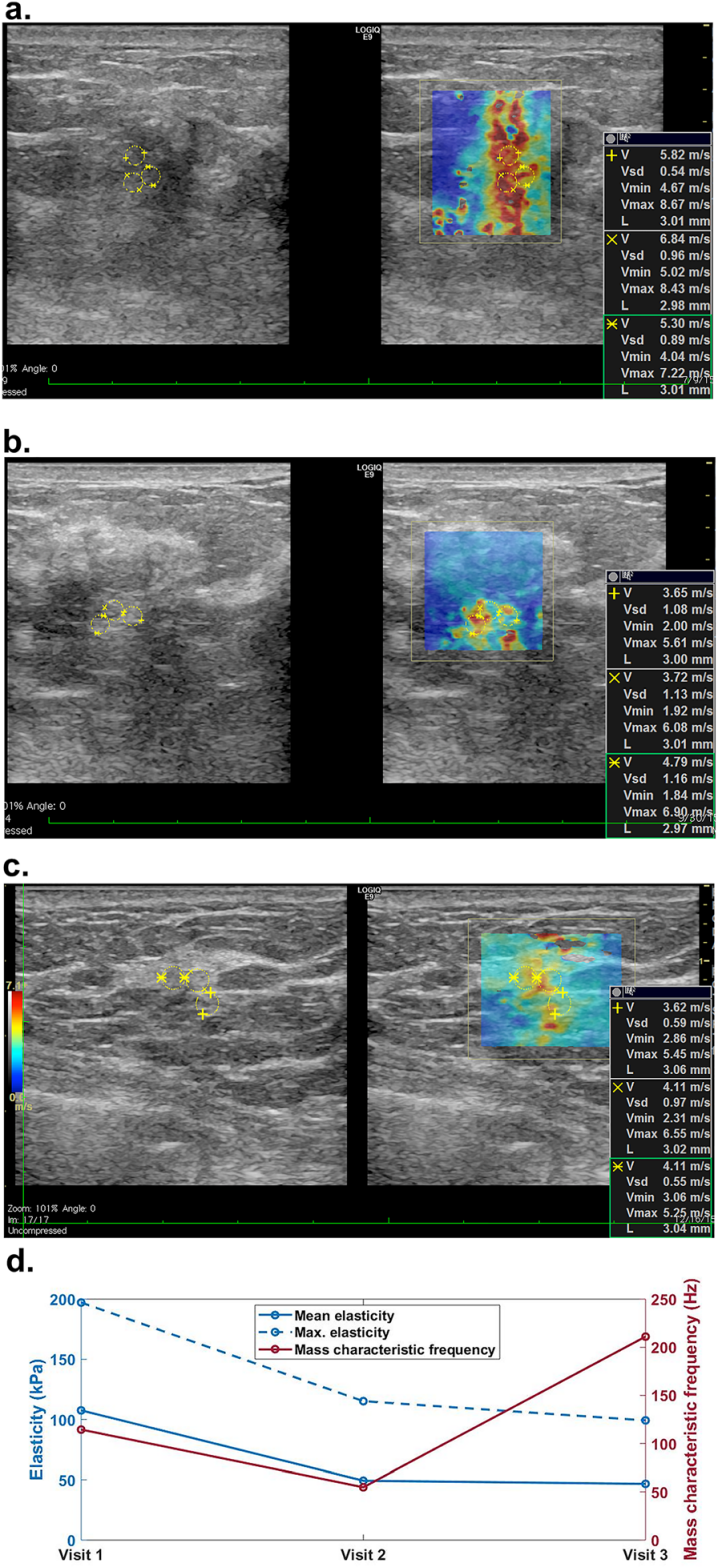
Shear wave speed map for a 48-year-old female with HER2+ tumor (grade III invasive ductal carcinoma) measured **a** before NACT, **b** during the mid-course of NACT, and **c** after NACT but before surgery. The mean elasticity, maximum elasticity, and mass characteristic frequency are shown in **d**. This is a responder with RCB score of 0. NACT, neoadjuvant chemotherapy; RCB, residual cancer burden

**Fig. 4:**
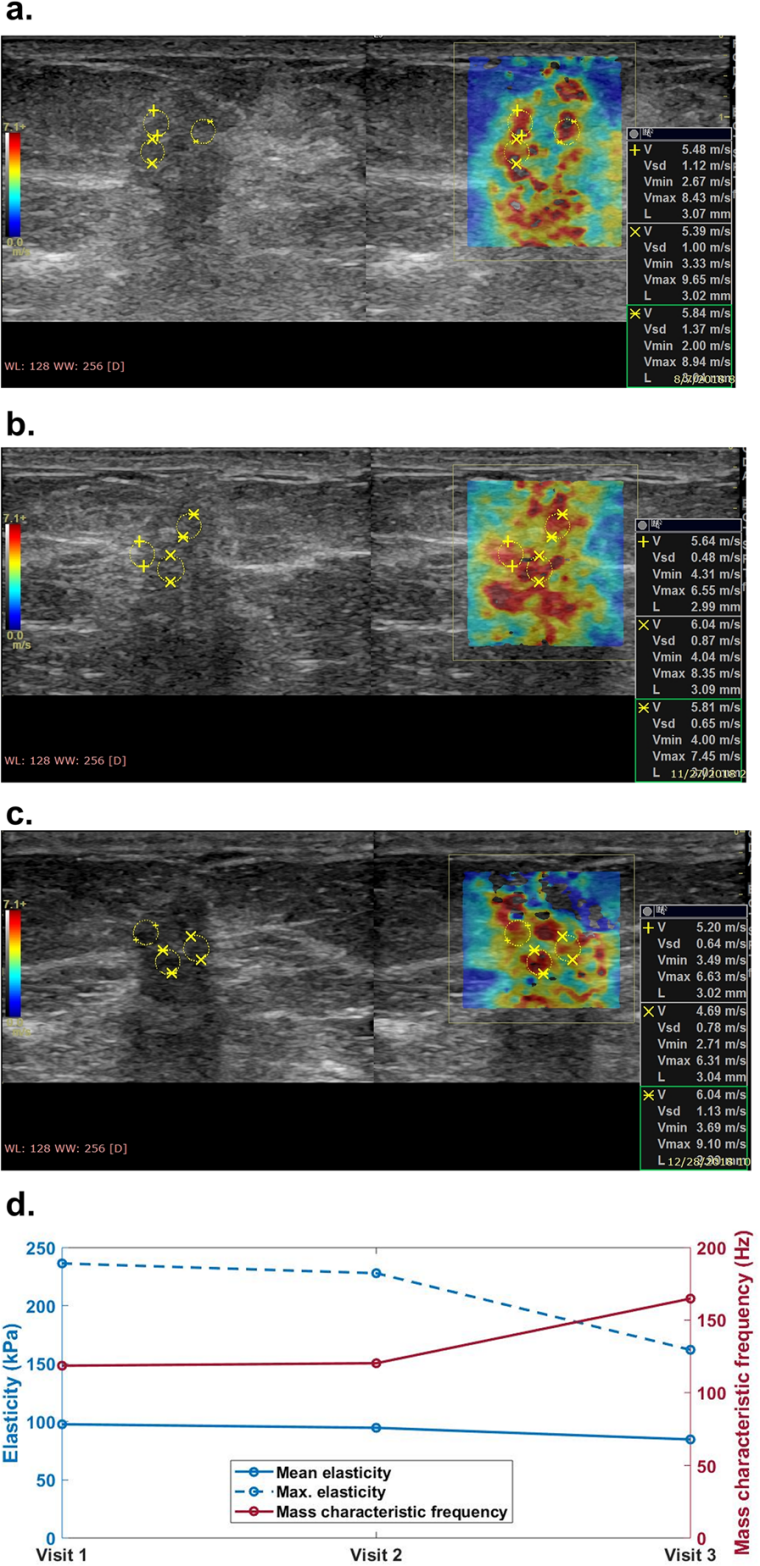
Shear wave speed map for a 59-year-old female with Luminal A tumor (grade II invasive ductal carcinoma) measured **a** before NACT, **b** during the mid-course of NACT, and **c** after NACT but before surgery. The mean elasticity, maximum elasticity, and mass characteristic frequency are shown in **d**. This is a non-responder with an RCB score of 1.6. NACT, neoadjuvant chemotherapy; RCB, residual cancer burden

**Fig. 5:**
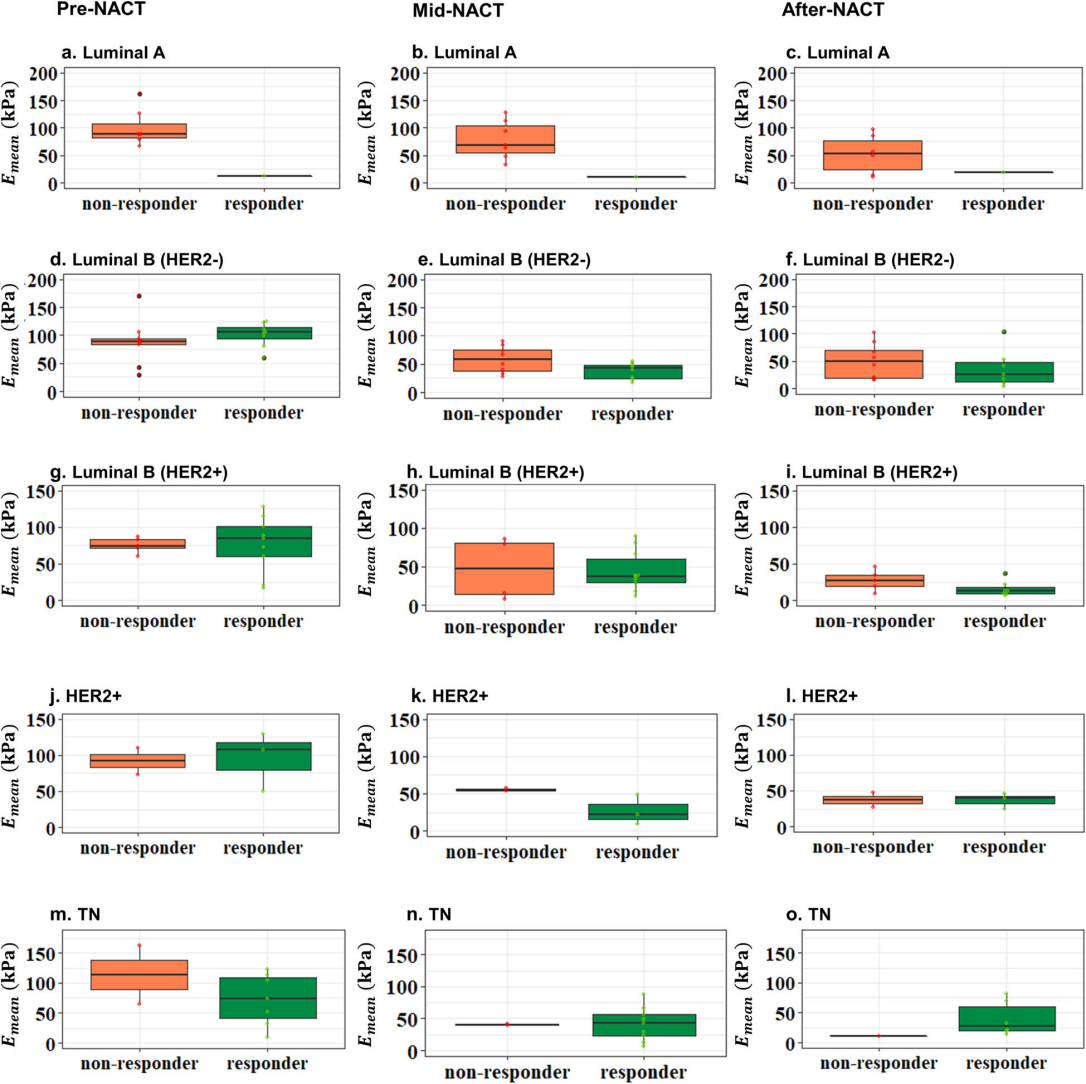
The mean shear wave elasticity measured before NACT, at the mid-course of NACT, and after NACT but before surgery for different molecular subtypes: **a**–**c** Luminal A type, **d**–**f** Luminal B (HER2-) type, **g**–**i** Luminal B (HER2+) type, **j**–**l** HER2+ type, and **m**–**o** TN type. NACT, neoadjuvant chemotherapy; TN, triple-negative

**Fig. 6:**
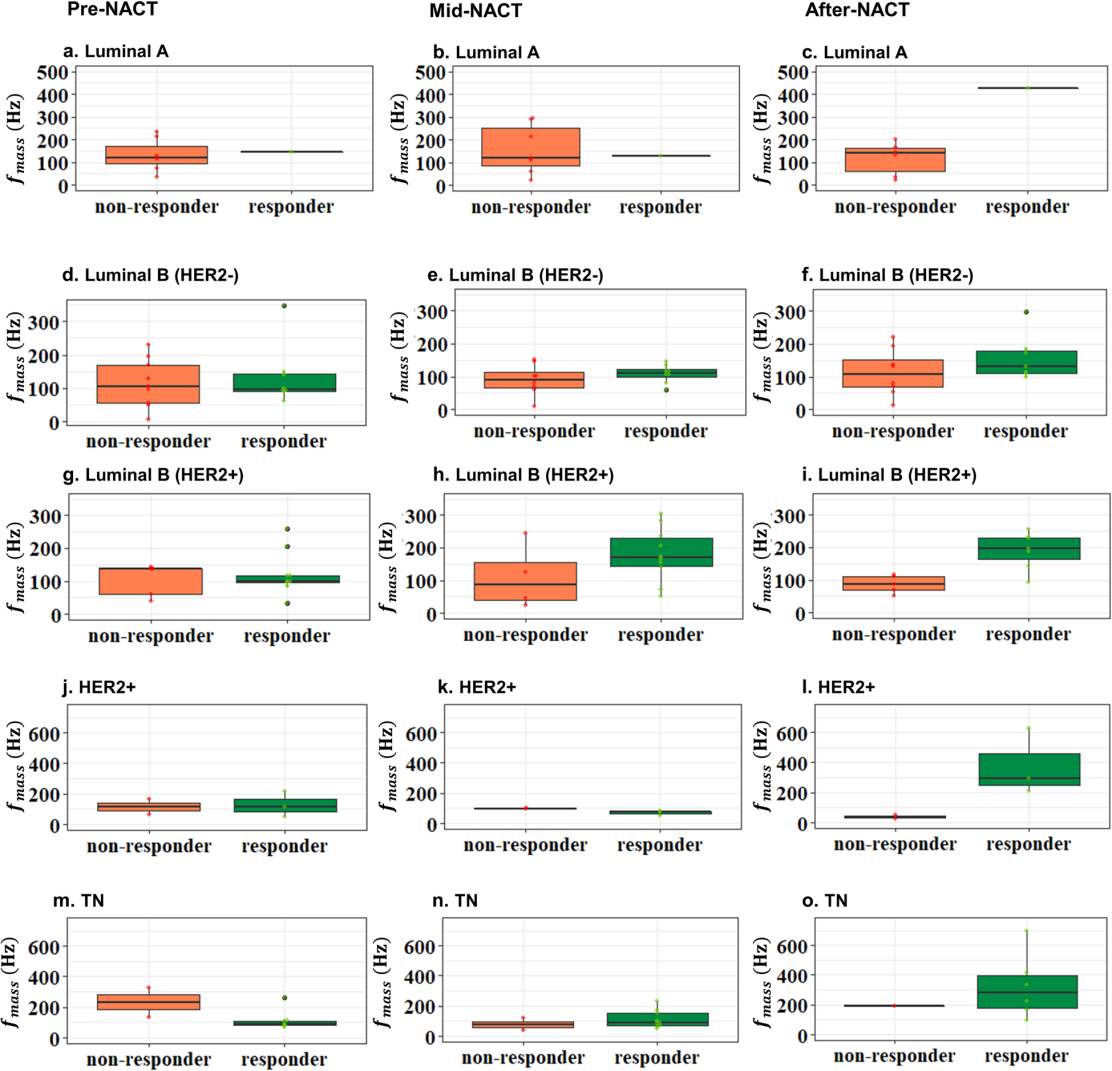
The mass characteristic frequency measured before NACT, at the mid-course of NACT, and after NACT but before surgery for different molecular subtypes: **a**–**c** Luminal A type, **d**–**f** Luminal B (HER2-) type, **g**–**i** Luminal B (HER2+) type, **j**–**l** HER2+ type, and **m**–**o** TN type. NACT, neoadjuvant chemotherapy; TN, triple-negative

For all three visits, non-responders showed higher averaged elasticity, elasticity ratio, and lower mass characteristic frequency. No significant difference was found in the change of elasticity, although there was a trend for the averaged change in stiffness in the responder group to be higher. A significant difference was found in the change of the mass characteristic frequency measured between the first and the third visits (*f*_mass1–3_, *p* < 0.001). Figures 3 and 4 show the typical SWS maps for a responder and a non-responder for the three SWE studies, respectively; *E*_mean_ and *f*_mass_ for different molecular subtypes measured during the three visits are shown in Figs. 5 and 6, respectively, indicating that stiffness decreased significantly for the responders, while remained high for the non-responders; the *f*_mass_ remained low for non-responders and increased significantly for responders.

### Leave-one-out cross-validation for the NACT response prediction

**Fig. 7:**
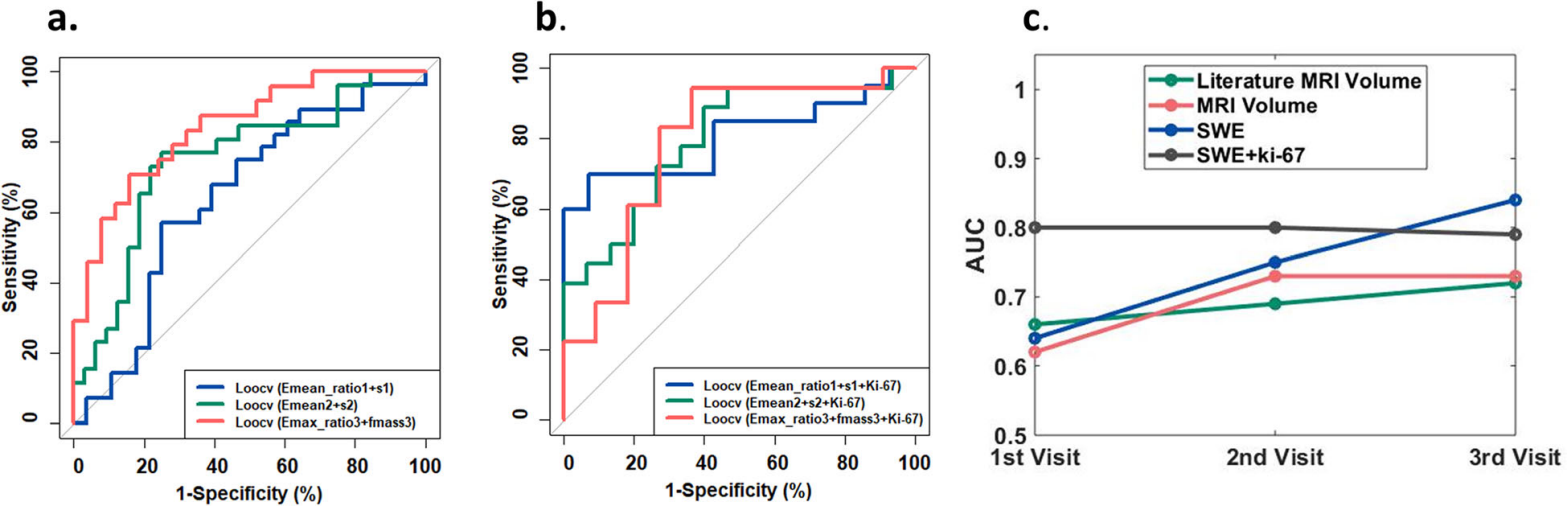
**a** ROCs for the three noninvasive models generated with the leave-one-out cross-validation, which is based on the combination of *E*_mean_ratio1_ and *s*_1_ for the first visit, the combination of *E*_mean2_ and *s*_2_ for the second visit, the combination of *E*_max_ratio3_ and *f*_mass3_ for the third visit. **b** ROCs for the three mixed models which were generated with the Ki-67 index added to the noninvasive models for ER-positive tumors. **c** Comparisons of the AUCs for the ROCs for NACT response prediction with the MRI volume from reference [23], the MRI volume in this study, the noninvasive models, and the mixed models. The 1st visit for the MRI volume measurement in [23] was after the first cycle of NACT treatment. ROC, receiver operating characteristic; SWE, shear wave elastography; PR, progesterone receptor; NACT, neoadjuvant chemotherapy; AUC, area under the curve; MRI, magnetic resonance imaging; IHC, immunohistochemical

Selected SWE parameters measured at each visit were combined using the LOOCV to predict the NACT treatment response, and the models were denoted as the noninvasive models. The *E*_mean_ratio1_ and *s*_1_ were combined for the first visit, the *E*_mean2_ and *s*_2_ were combined for the second visit, and the *E*_max_ratio3_ and *f*_mass3_ were combined for the third visit. The corresponding ROCs are shown in Fig. 7a. The AUCs, optimal cutoffs, and the corresponding specificity and sensitivity are summarized in Table 3. The AUC (0.84, 0.95 CI 0.78–0.97) for the third visit was the highest among the three visits. A significant difference was found for the AUC between the first and the third visits (*p* = 0.04). No significant difference was found between the second and the third visits (*p* = 0.29). Therefore, the AUC (0.75, 0.95 CI 0.62–0.88) for the second visit gave the second best prediction, given the balance between timing and accuracy.

**Table 3:** Summary of the ROCs for the leave-one-out cross-validation analysis

Parameters	AUC	Cutoff	Specificity	Sensitivity
Noninvasive model				
The first visit	0.64 (0.95 CI 0.49–0.79)	0.51	0.75	0.57
The second visit	0.75 (0.95 CI 0.62–0.88)	0.38	0.75	0.77
The third visit	0.84 (0.95 CI 0.78–0.97)	0.58	0.84	0.71
Mixed model				
The first visit	0.80 (0.95 CI 0.65–0.96)	0.68	0.93	0.70
The second visit	0.80 (0.95 CI 0.64–0.95)	0.53	0.73	0.72
The third visit	0.79 (0.95 CI 0.60–0.98)	0.62	0.73	0.83

*AUC* area under the curve, *CI* confidence interval, *ROC* receiver operating characteristic

For ER-positive tumors, the Ki-67 index obtained from the pre-NACT biopsy was then added to the selected SWE parameters included in the noninvasive models with the LOOCV to predict NACT response. The corresponding models were denoted as the mixed models, with ROCs shown in Fig. 7b. The AUCs, optimal cutoffs, and the corresponding specificity and sensitivity are summarized in Table 3. Among the mixed models, the ROC for the first visit showed the best prediction (0.80, 0.95 CI 0.65–0.96). With an optimal of 0.68, the specificity was 0.93 and the sensitivity was 0.70.

The AUCs for both the invasive models and the mixed models during the three visits are compared in Fig. 7c. The AUCs for the NACT response prediction with the MRI volume during the three visits were also plotted. The corresponding AUCs were 0.62 (0.95 CI 0.45–0.78), 0.73 (0.95 CI 0.46–1.00), and 0.73 (0.95 CI 0.54–0.92), respectively. No significant difference was found among the three AUCs. Since most patients only had the first MRI imaging before NACT, the number of MRI volume results available for the second and third visit was relatively small. Therefore, we also included the AUCs of the MRI volume adapted from [24] for reference. In contrast to the calculation method used in this study, tumor volume in [24] was computed by summing all voxels with percentage enhancement (PE) above a nominal threshold value of 70%, and *PE* = ((*S*_1_*-S*_0_)/*S*_0_) × 100%, where *S*_0_, *S*_1_, and *S*_2_ represented the signal intensities on the precontrast, early postcontrast, and late postcontrast images, respectively.

## Discussion

The results of our study demonstrate that SWE aids accurate assessment and early prediction of tumor response to NACT; for ER-positive tumors, combining the Ki-67 index with some SWE parameters can further improve the response prediction. Our study showed that the averaged stiffness decreased for both the responders and the non-responders. Moreover, the changes in stiffness for both the *E*_mean_ and *E*_max_ between the first two visits were larger than the changes between the second and the third visits. The slower drop in stiffness during the later course of NACT treatment may be due to hypoxia, which is associated with increased matrix stiffness in the non-necrotic area of the tumors [29, 30]. Moreover, it has been shown that residual tumor appears to be stiffer than the fibrous tissue left for the pCR [22]. These changes in stiffness can also be detected with SWE during the NACT treatment [8, 31].

The *f*_mass_ is a newly introduced SWE parameter for breast cancer characterization, and a lower *f*_mass_ value is significantly correlated with poor histologic prognostic factors. The average *f*_mass_ value for the non-responders stayed relatively constant throughout the NACT treatment. Recalling the definition of *f*_mass_, this result indicated that the change of SWS and mass size during the NACT was such that the net effect on *f*_mass_ was minimal for the non-responders. This study showed that the averaged *f*_mass_ for the responders was higher than that for the non-responders during all three visits. For the third visit, a significant difference was found among the responders and non-responders. Among the responders, there was no significant difference between *f*_mass1_ and *f*_mass2_. When compared to the average *f*_mass1_, the average *f*_mass3_ significantly increased by 70%, indicating that the effect of size reduction was dominant on *f*_mass_ (compared to the change of minimum SWS) after the mid-course of NACT. A similar trend was observed in each molecular subtype. However, the average *E*_mean_ decreased continuously throughout the NACT. When compared to the first measurement, the average *E*_mean_ decreased by 48% during the second visit and by 62% during the third visit. Therefore, *f*_mass_ could be used as a useful indicator to determine the end-of-treatment point for the NACT for responders. Currently, response to NACT is routinely assessed using MRI, clinical ultrasound, and mammography [22]. The addition of *f*_mass_ could further facilitate the individualized NACT treatment plan. We plan to extend our study to a larger number of patients and with more frequent *f*_mass_ measurements in the course of NACT to further investigate the role of *f*_mass_ in predicting earlier response to NACT.

Predictions of response to NACT with the noninvasive models which combine the SWE parameters show promising results for all three visits. To balance the timing and accuracy, the parameters obtained during the mid-course of NACT could be used for evaluating the NACT treatment outcome. Similarly, a previous study showed that the optimal time for early evaluation to identify patients who would be responsive to treatment was after 2 cycles of treatment and immediately before the third cycle of the therapy [29].

The Ki-67 index is an important predictive factor for the effectiveness of NACT [32, 33]. Breast cancer with a high Ki-67 index level has repeatedly been shown to respond better to chemotherapy. A previous study also showed that there was no pathological complete response in cases with Ki-67 < 25% [34]. In this study, we found that the average Ki-67 value was higher in responders than in non-responders. Mixed models, which combined the Ki-67 index obtained before NACT with the noninvasive models, were proposed for ER-positive tumors. When compared to the noninvasive model for the second visit, an earlier prediction with improved accuracy could be achieved with the mixed model. Similarly, the study by Ma et al. has shown the potential value of adding Ki-67 to shear wave parameters for better and earlier prediction of the response to therapy [35].

Both the noninvasive models and the mixed models were generated with the leave-one-out cross-validation analysis, which included internal validations to quantify any optimism in the predictive performance and adjust the models for overfittings [36, 37]. Therefore, the models proposed in this paper for predicting the response to NACT have high reproducibility and stability.

Studies showed that MRI volumetric assessment was more accurate than the diameter for earlier detection of treatment response [22, 24]. The volume from clinical MRI was also recorded in this study for all three visits. Though the MRI volume data was limited in this study, the ROCs were comparable to the results from a previous study with a larger patient number. Moreover, both the AUCs from the noninvasive model for the second visit and from the mixed model for the first visit were higher than the MRI volume prediction at the corresponding time point. However, the results of proposed prediction models could vary among different study populations. Therefore, a future study based on a larger population will be helpful for comparing the results from the MRI prediction and the proposed models in this study.

There are some limitations in this study. Firstly, the sample size was relatively small; moreover, as shown in the flowchart, some patients did not complete all three visits for the SWE studies, leading to some missing data. However, the leave-one-out cross-validation has been applied to compensate for the sample number limitation. Secondly, this is a one-center study. Thus, a multicenter study with a larger population is required to further investigate the role of SWE parameters in NACT response prediction.

In summary, this study investigated the application of SWE in discriminating the responders from non-responders to chemotherapy, before NACT, during the mid-course of NACT, and after NACT but prior to surgery. In conclusion, when the noninvasive model is used for response prediction, a balance between the timing and accuracy is achieved when the *E*_mean2_ and *s*_2_ are measured during the mid-course of the treatment. For ER-positive tumors, an even earlier and more accurate response prediction could be obtained with the combination of Ki-67 index, *E*_mean_ratio1_, and *s*_1_ measured before the treatment using the mixed model. Moreover, this study also shows that *f*_mass_ is useful in determining the endpoint of the NACT.

## Conclusions

Our study findings highlight the value of SWE estimation in the mid-course of NACT for the early prediction of treatment response. For ER+ tumors, the addition of Ki-67 improves the predictive power of SWE. Moreover, *f*_mass_ is presented as a new marker in predicting the endpoint of NACT in responders. These results may facilitate personalizing the treatment regimens of patients with breast cancer receiving NACT. Furthermore, the role of these SWE parameters can be validated in the future by carrying out a multicenter prospective study with a larger patient population.

## Data Availability

The datasets used and/or analyzed during the current study are available from the corresponding author on reasonable request.

## Abbreviations

ASCO: American Society of Clinical Oncology
CAP: College of American Pathologists
CI: Confidence interval
ECM: Extracellular matrix
ER: Estrogen receptor
FISH: Fluorescence in situ hybridization
HER2: Human epidermal growth factor receptor 2
IHC: Immunohistochemical
LOOCV: Leave-one-out cross-validation
MRI: Magnetic resonance imaging
NACT: Neoadjuvant chemotherapy
pCR: Pathological complete response
PR: Progesterone receptor
RCB: Residual cancer burden
ROC: Receiver operating characteristic
ROIs: Regions of interest
SWE: Shear wave elastography
SWS: Shear wave speed
US: Ultrasound
AUC: Area under the curve

## References

[1] VAN J, HAGE C, VELDE JJ, Tubiana-Hulin M, Vandervelden C: Preoperative chemotherapy in primary operable breast cancer: results from the European Organization for Research and Treatment of Cancer trial 10902. Impact of age, tumor characteristics, and treatment on local control and disease outcome in early stage breast cancer 2001:33. 10.1200/JCO.2001.19.22.4224 11709566

[2] Fisher B, Bryant J, Wolmark N, Mamounas E, Brown A, Fisher ER, Wickerham DL, Begovic M, DeCillis A, Robidoux A (1998). Effect of preoperative chemotherapy on the outcome of women with operable breast cancer. J Clin Oncol.

[3] Kim C, Han S-A, Won KY, Hong IK, Kim DY (2020). Early prediction of tumor response to neoadjuvant chemotherapy and clinical outcome in breast cancer using a novel FDG-PET parameter for cancer stem cell metabolism. J Person Med.

[4] Magbanua MJM, Hendrix LH, Hyslop T, Barry WT, Winer EP, Hudis C, Toppmeyer D, Carey LA, Partridge AH, Pierga J-Y: Abstract A50: circulating tumor DNA (ctDNA) and magnetic resonance imaging (MRI) for monitoring and predicting response to neoadjuvant therapy (NAT) in high-risk early breast cancer patients in the I-SPY 2 TRIAL. In. Miami: AACR; 2020.

[5] Tudorica A, Oh KY, Chui SY, Roy N, Troxell ML, Naik A, Kemmer KA, Chen Y, Holtorf ML, Afzal A (2016). Early prediction and evaluation of breast cancer response to neoadjuvant chemotherapy using quantitative DCE-MRI. Transl Oncol.

[6] Cho N, Im S-A, Park I-A, Lee K-H, Li M, Han W, Noh D-Y, Moon WK (2014). Breast cancer: early prediction of response to neoadjuvant chemotherapy using parametric response maps for MR imaging. Radiology.

[7] Li Q, Xiao Q, Li J, Wang Z, Wang H, Gu Y (2020). Value of machine learning with CE-MRI radiomics for early prediction of pathological complete response to neoadjuvant therapy in HER2-positive invasive breast cancer.

[8] Evans A, Armstrong S, Whelehan P, Thomson K, Rauchhaus P, Purdie C, Jordan L, Jones L, Thompson A, Vinnicombe S (2013). Can shear-wave elastography predict response to neoadjuvant chemotherapy in women with invasive breast cancer?. Br J Cancer.

[9] Lee SH, Chang JM, Han W, Moon H-G, Koo HR, Gweon HM, Kim WH, Noh D-Y, Moon WK (2015). Shear-wave elastography for the detection of residual breast cancer after neoadjuvant chemotherapy. Ann Surg Oncol.

[10] Chagpar AB, Middleton LP, Sahin AA, Dempsey P, Buzdar AU, Mirza AN, Ames FC, Babiera GV, Feig BW, Hunt KK (2006). Accuracy of physical examination, ultrasonography, and mammography in predicting residual pathologic tumor size in patients treated with neoadjuvant chemotherapy. Ann Surg.

[11] Chang JM, Moon WK, Cho N, Yi A, Koo HR, Han W, Noh D-Y, Moon H-G, Kim SJ (2011). Clinical application of shear wave elastography (SWE) in the diagnosis of benign and malignant breast diseases. Breast Cancer Res Treat.

[12] Bai M, Du L, Gu J, Li F, Jia X (2012). Virtual touch tissue quantification using acoustic radiation force impulse technology: initial clinical experience with solid breast masses. J Ultrasound Med.

[13] Denis M, Bayat M, Mehrmohammadi M, Gregory A, Song P, Whaley DH, Pruthi S, Chen S, Fatemi M, Alizad A (2015). Update on breast cancer detection using comb-push ultrasound shear elastography. IEEE T Ul Transon Ferr.

[14] Evans A, Whelehan P, Thomson K, McLean D, Brauer K, Purdie C, Jordan L, Baker L, Thompson A (2010). Quantitative shear wave ultrasound elastography: initial experience in solid breast masses. Breast Cancer Res.

[15] Golatta M, Schweitzer-Martin M, Harcos A, Schott S, Gomez C, Stieber A, Rauch G, Domschke C, Rom J, Schütz F (2014). Evaluation of virtual touch tissue imaging quantification, a new shear wave velocity imaging method, for breast lesion assessment by ultrasound. Biomed Res Int.

[16] Zhu J, Xiong G, Trinkle C, Xu R (2014). Integrated extracellular matrix signaling in mammary gland development and breast cancer progression. Histol Histopathol.

[17] Cox TR, Erler JT (2011). Remodeling and homeostasis of the extracellular matrix: implications for fibrotic diseases and cancer. Dis Model Mech.

[18] Pickup MW, Mouw JK, Weaver VM (2014). The extracellular matrix modulates the hallmarks of cancer. EMBO Rep.

[19] Gilkes DM, Bajpai S, Wong CC, Chaturvedi P, Hubbi ME, Wirtz D, Semenza GL (2013). Procollagen lysyl hydroxylase 2 is essential for hypoxia-induced breast cancer metastasis. Mol Cancer Res.

[20] Jing H, Cheng W, Li Z-Y, Ying L, Wang Q-C, Wu T, Tian J-W (2016). Early evaluation of relative changes in tumor stiffness by shear wave elastography predicts the response to neoadjuvant chemotherapy in patients with breast cancer. J Ultrasound Med.

[21] Evans A, Whelehan P, Thompson A, Purdie C, Jordan L, Macaskill J, Waugh S, Fuller-Pace F, Brauer K, Vinnicombe S (2018). Prediction of pathological complete response to neoadjuvant chemotherapy for primary breast cancer comparing interim ultrasound, shear wave elastography and MRI. Eur J Ultrasound.

[22] Evans A, Whelehan P, Thompson A, Purdie C, Jordan L, Macaskill J, Henderson S, Vinnicombe S (2018). Identification of pathological complete response after neoadjuvant chemotherapy for breast cancer: comparison of greyscale ultrasound, shear wave elastography, and MRI. Clin Radiol.

[23] Symmans WF, Peintinger F, Hatzis C, Rajan R, Kuerer H, Valero V, Assad L, Poniecka A, Hennessy B, Green M (2007). Measurement of residual breast cancer burden to predict survival after neoadjuvant chemotherapy. J Clin Oncol.

[24] Hylton NM, Blume JD, Bernreuter WK, Pisano ED, Rosen MA, Morris EA, Weatherall PT, Lehman CD, Newstead GM, Polin S (2012). Locally advanced breast cancer: MR imaging for prediction of response to neoadjuvant chemotherapy—results from ACRIN 6657/I-SPY TRIAL. Radiology.

[25] Wolff AC, Hammond MEH, Schwartz JN, Hagerty KL, Allred DC, Cote RJ, Dowsett M, Fitzgibbons PL, Hanna WM, Langer A (2007). American Society of Clinical Oncology/College of American Pathologists guideline recommendations for human epidermal growth factor receptor 2 testing in breast cancer. Arch Pathol Lab Med.

[26] Goldhirsch A, Wood WC, Coates AS, Gelber RD, Thürlimann B, Senn H-J, Members P (2011). Strategies for subtypes—dealing with the diversity of breast cancer: highlights of the St Gallen International Expert Consensus on the Primary Therapy of Early Breast Cancer 2011. Ann Oncol.

[27] Denis M, Gregory A, Bayat M, Fazzio RT, Whaley DH, Ghosh K, Shah S, Fatemi M, Alizad A: Correlating tumor stiffness with immunohistochemical subtypes of breast cancers: prognostic value of comb-push ultrasound shear elastography for differentiating luminal subtypes. PLoS One. 2016;11(10):1–14. 10.1371/journal.pone.0165003. 10.1371/journal.pone.0165003 PMC5077080 27776153

[28] van der Laan M, Polley E, Hubbard A: Super learner. Statistical applications of genetics and molecular biology, 6, article 25. In.; 2007. 10.2202/1544-6115.1309 17910531

[29] Zhang J, Tan X, Zhang X, Kang Y, Li J, Ren W, Ma Y (2020). Efficacy of shear-wave elastography versus dynamic optical breast imaging for predicting the pathological response to neoadjuvant chemotherapy in breast cancer. Eur J Radiol.

[30] Le-Frère-Belda M-A, Latorre-Ossa H, Fitoussi V, Redheuil A, Assayag F, Pidial L, Gennisson J-L, Tanter M, Cuénod C-A, Fournier LS (2016). Supersonic shear wave elastography of response to anti-cancer therapy in a xenograft tumor model. Ul Trasound Med Biol.

[31] Ma Y, Zhang S, Li J, Li J, Kang Y, Ren W (2017). Comparison of strain and shear-wave ultrasounic elastography in predicting the pathological response to neoadjuvant chemotherapy in breast cancers. Eur Radiol.

[32] Asselain B, Barlow W, Bartlett J, Bergh J, Bergsten-Nordström E, Bliss J, Boccardo F, Boddington C, Bogaerts J, Bonadonna G (2018). Long-term outcomes for neoadjuvant versus adjuvant chemotherapy in early breast cancer: meta-analysis of individual patient data from ten randomised trials. Lancet Oncol.

[33] Jain P, Doval DC, Batra U, Goyal P, Bothra SJ, Agarwal C, Choudhary DK, Yadav A, Koyalla VPB, Sharma M (2019). Ki-67 labeling index as a predictor of response to neoadjuvant chemotherapy in breast cancer. Jpn J Clin Oncol.

[34] Nishimura R, Osako T, Okumura Y, Hayashi M, Arima N (2010). Clinical significance of Ki-67 in neoadjuvant chemotherapy for primary breast cancer as a predictor for chemosensitivity and for prognosis. Breast Cancer.

[35] Ma Y, Zhang S, Zang L, Li J, Li J, Kang Y, Ren W (2016). Combination of shear wave elastography and Ki-67 index as a novel predictive modality for the pathological response to neoadjuvant chemotherapy in patients with invasive breast cancer. Eur J Cancer.

[36] Moons KG, Altman DG, Reitsma JB, Ioannidis JP, Macaskill P, Steyerberg EW, Vickers AJ, Ransohoff DF, Collins GS (2015). Transparent Reporting of a multivariable prediction model for Individual Prognosis or Diagnosis (TRIPOD): explanation and elaboration. Ann Intern Med.

[37] Collins GS, Reitsma JB, Altman DG, Moons KG (2015). Transparent reporting of a multivariable prediction model for individual prognosis or diagnosis (TRIPOD) the TRIPOD statement. Circulation.

